# Hidden faces of alpha-synuclein: Cryo-EM revelation of fibril polymorphs driven by disease, mutations, and PTMs

**DOI:** 10.1016/j.bbadva.2025.100179

**Published:** 2025-12-14

**Authors:** Mitra Pirhaghi, Fatemeh Mamashli, Bagher Davaeil, Mahya Mohammad-Zaheri, Zahra Mousavi-Jarrahi, Jörg Tatzelt, Ali Akbar Saboury

**Affiliations:** a Department of Biological Sciences, Institute for Advanced Studies in Basic Sciences (IASBS), Zanjan 6673145137, Iran; b Department Biochemistry of Neurodegenerative Diseases, Institute of Biochemistry and Pathobiochemistry, Ruhr University Bochum, Bochum 44801, Germany; c Institute of Biochemistry and Biophysics, University of Tehran, Tehran 1417614335, Iran

**Keywords:** Amyloid fibril, α-Synuclein, Parkinson's disease, Fibril polymorphism, Cryo-EM

## Abstract

Alpha-synuclein (α-Syn) is a neuronal protein implicated in the pathogenesis of several neurodegenerative disorders collectively known as synucleinopathies, including Parkinson’s disease, dementia with Lewy bodies, and multiple system atrophy. This article provides a comprehensive overview of the structural characteristics of α-Syn, emphasizing its fibrillar aggregation and the resulting polymorphic fibril forms. Using advances in cryo-electron microscopy, a diverse range of α-Syn fibril polymorphs has been elucidated, both from *in vitro* preparations and patient-derived brain samples. We further explore the impact of mutation and post-translational modifications—such as truncation and phosphorylation —on α-Syn structure and the unique polymorphs they induce. The article underscores the biological and pathological relevance of α-Syn polymorphism, highlighting how different structural strains may underlie the structural and pathological heterogeneity observed in synucleinopathies. Understanding the mechanisms driving polymorph formation is critical for deciphering disease progression and developing targeted therapeutic strategies.

## Highlights

• Cryo-EM structures reveal extensive fibril polymorphism in α-synuclein.
• Patient-derived fibrils show distinct architectures linked to synucleinopathies.
• Mutations and Post-translational modifications drive unique α-syn fibril conformations.
• Structural polymorphs provide insight into mechanisms of disease heterogeneity.

**Abbreviations**
α-Synuclein: α-Syn
Fourier-Transform Infrared Microscopy: FTIR
Lewy Bodies: LBs
Dementia with Lewy Bodies: DLB
Lewy body disease: LBD
Multiple System Atrophy: MSA
Non-Amyloid β Component: NAC
Protein Misfolded Cyclic Amplification: PMCA
Parkinson’s Disease: PD
PD Dementia: PDD
Small-Angle X-Ray Scattering: SAXS
solid-state NMR spectroscopy: ssNMR
cryo-Electron Microscopy: cryo-EM
Vesicle-Associated Membrane Protein 2: VAMP2
Electron Microscopy: EM
cryo-Electron Microscopy: cryo-EM
Atomic Force Microscopy: AFM
Circular Dichroism Spectroscopy: CD
Electron Paramagnetic Resonance Spectroscopy: EPR
micro-Electron Diffraction: micro-ED
Scanning Force Microscopy: SFM
Scanning Transmission Electron Microscopy: STEM
MSA-amplified fibrils: MSA-AF
PD-amplified fibrils: PD-AF
Cerebrospinal Fluid: CSF
Preclinical PD: pre-PD
Late-Stage Postmortem PD: post-PD
Lewy Neurites: LNs
Glial Cytoplasmic Inclusions: GCIs
Molecular Dynamics: MD
Post-Translational Modifications: PTMs
Wild-Type: WT
Glycosaminoglycans: GAGs
The Cerebrospinal Fluid: CSF
Juvenile-Onset Synucleinopathy: JOS
Phosphate-Buffered Saline: PBS

## 1 Introduction

**Fig. 1:**
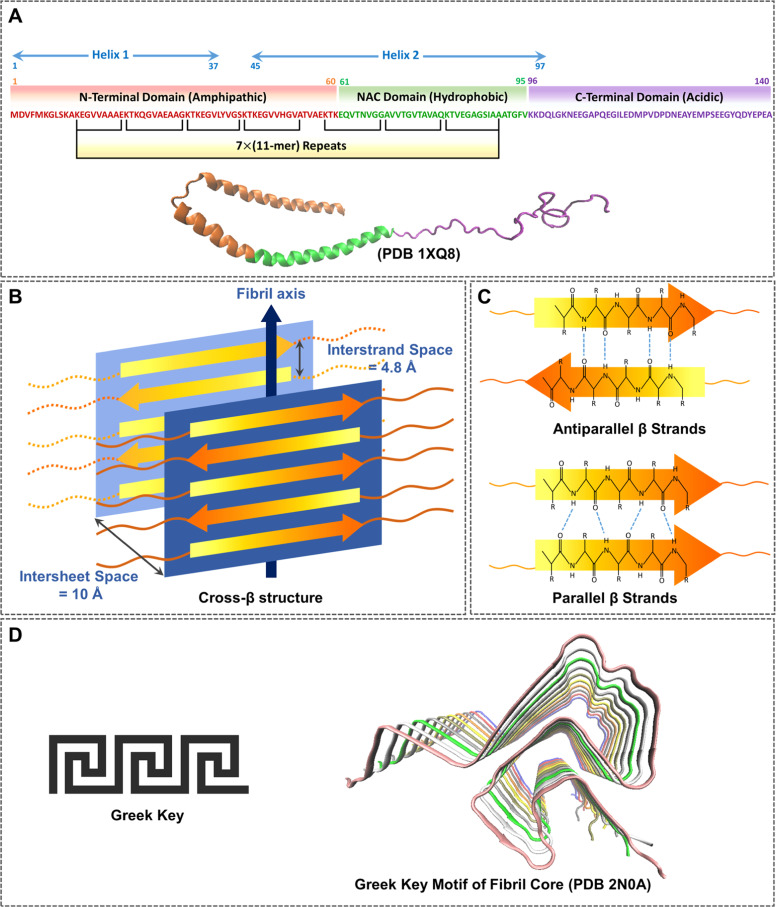
**The structure of α-Syn and amyloid cross-β structure. (A)** Schematic representation of the primary sequence of α-Syn with three distinct domains: the N-terminal domain (**red**) contains a lipid-binding amphipathic motif, the central NAC domain (**green**) contains a stretch of hydrophobic residues, and the C-terminal domain (**purple**) is rich in acidic amino acids. The lipid binding domain can be divided into seven highly conserved 11-mer sequences. Upon binding to lipid membranes, the N-terminal and NAC domains fold into two amphipathic helices (helix 1 and helix 2); the C-terminal tail does not contribute to membrane binding. **(B)** An amyloid cross-β structure is characterized by a 4.7 Å inter-strand distance and an approximately 10 Å inter-sheet distance. **(C)** Antiparallel or parallel arrangement of β-strands. **(D)** Greek key motif of the fibrillar core of α-Syn.

The pathological misfolding and aggregation of α-synuclein (α-Syn) are fundamental mechanisms in the onset of several neurodegenerative diseases, including Parkinson’s disease (PD), dementia with Lewy bodies (DLB), Parkinson’s disease dementia (PDD), and multiple system atrophy (MSA), collectively known as synucleinopathies [[1], [2], [3], [4]]. α-Syn is a 140-residue intrinsically disordered protein composed of three domains [[5], [6], [7]]. The amphipathic N-terminal domain (residues 1‒60) containing positive charges, the central hydrophobic region (residues 61‒95) called the non-amyloid β component (NAC) domain, and the acidic C-terminal domain (residues 96‒140) containing negative charges (Fig. 1) [[8], [9], [10]]. Within residues 10–98 of α-Syn, there are seven imperfect repeats of the XKTKEGVXXXX sequence, with the X positions mainly consisting of hydrophobic residues [11,12]. This stretch can form a lipid-binding α-helix [13,14]. Under physiological conditions, α-Syn primarily exists as unstructured monomers, forming α-helices upon binding to synaptic vesicles or plasma membranes [9,10,15,16]. In pathological states, it adopts a β-sheet-rich structure, leading to amyloid fibril formation [9,[17], [18], [19]].

Even within a single sample, amyloid fibrils can exhibit polymorphism, where a single peptide or protein forms various distinct, self-propagating fibrillar structures [20]. Biochemical and structural studies on cell lines, animal models, and human brain extracts have recently provided initial evidence that structural differences in amyloid fibrils may explain the observed disease variations [[21], [22], [23], [24], [25], [26], [27]]. The presence of polymorphs has been associated with the strain phenomenon in prions, where different PrP strains correlate with various clinical phenotypes observed in prion diseases [[28], [29], [30]]. Prion strains display different protease resistance, glycosylation profiles, electrophoretic mobility, and seeding abilities *in vitro*. Consequently, they are marked by distinct clinical signs, lesion characteristics, disease onset, and incubation periods *in vivo* [[31], [32], [33]]. Increasing evidence suggests that α-Syn also exhibits prion-like strain phenomena, accounting for its link to various neurodegenerative diseases with distinct clinical and pathological phenotypes [27,[34], [35], [36]].

Recent advances in solid-state NMR spectroscopy (ssNMR) and cryo-electron microscopy (cryo-EM) have provided deeper insights into the α-Syn fibrillar polymorphisms at the molecular level [[37], [38], [39], [40], [41]]. Fibril polymorphs can be identified by their diameter, presence of twists, number and packing of protofilaments, side-chain interactions, and the secondary and tertiary structure arrangements [39,[42], [43], [44]]. Numerous studies have confirmed that different α-Syn strains contribute to the clinical variations observed in synucleinopathies, probably elucidating the link between α-Syn aggregates and disease heterogeneity [[24], [25], [26], [27],[45], [46], [47]]. Despite advancements, the origin of polymorphism *in vivo* and the factors regulating strain formation remain unclear [48].

This review explores the polymorphic characteristics of α-Syn, its implications, and the reasons behind fibril polymorphism. It covers the cross-β structure of α-Syn fibrils, evidence of polymorphism, various aggregation mechanisms, and the principles and significance of these polymorphisms. Structural insights into α-Syn amyloids are crucial for understanding the cytotoxicity of amyloid aggregates and the molecular mechanisms of synucleinopathies.

## 2 Fibril formation by α-Synuclein

As an intrinsically disordered protein, α-Syn exhibits various conformational states [49]. β-sheet enriched conformations may become incidentally favored and stabilized [50]. The nucleation of these structures forms "seeds" that initiate the templated growth of amyloid assemblies, consuming neuronal α-Syn. These fibrils are partially sequestered into Lewy bodies (LBs) [51], while a significant fraction remains soluble or transmissible, spreading between neurons and driving prion-like propagation of amyloid assemblies [52,53]. During amyloid stacking, α-Syn can adopt multiple structural folds, resulting in the emergence of fibril polymorphs [[37], [38], [39],^42^,[54], [55], [56]]. These polymorphs inherit and propagate their supramolecular characteristics through templated growth [21,23,57,58].

Protein amyloids are generally enriched in a cross-β structure. A cross-β structure is ribbon-like, with β-sheets extending along the fibril's length, and β-strands oriented approximately perpendicular to the direction of fibril growth, linked by inter-strand backbone hydrogen bonds aligned nearly parallel to the growth direction (Fig. 1B) [59]. The relative cross-β structure determines whether two β-strands are arranged in an antiparallel or parallel manner (Fig. 1C). The presence of cross-β structures in amyloid fibrils was initially identified through X-ray fibril diffraction [60] and is further supported by electron diffraction [61], electron microscopy (EM) [62] and ssNMR [63].

## 3 3D structure of α-Syn fibrils

The first high-resolution 3D structure of α-Syn fibrils was obtained using high-resolution cryo-EM [64]. This structure obtained from ∼5 nm diameter single protofilaments, contrasts with the two protofilaments with ∼4 nm width. They discovered a unique structural composition, in which the core residues were configured into β-serpentine, parallel β-sheets with a Greek-key topology (Fig. 1D). This fold demonstrates hydrogen bonds oriented along the fibril axis, which are perpendicular to the hydrogen bond geometry found in a standard Greek-key motif. The innermost β-sheet of the core, containing residues 71–82, is crucial for fibril formation. The Greek-key morphology of α-Syn is densely packed with small amino acid residues, such as Gly, Ala, and Ser, in the fibrillar core. They also identified the presence of salt bridges (E46-K80), a glutamine ladder (Q79), and hydrophobic packing of aromatic residues (F94) within the hydrophobic core of a single fibril filament, which stabilize the in-register β-sheet [64].

Li et al. [65] applied cryo-EM to determine the full-length α-Syn fibrillar structure. The structure exhibited a left-handed helix with a helical pitch of 239 nm and a periodicity of 120 ± 14 nm (half pitch). In each fibril, the helical pitch includes two protofilaments. Residues 50–57 (^50^HGVATVAE^57^) from one protofilament make a dimer interface with opposing α-Syn subunits via a steric-zipper. The steric-zipper is stabilized by hydrophobic interactions between A53 and V55. The electrostatic interactions between K58-E61 and E46-K80 play a significant role in folding topology. Regarding the details revealed by Li et al. [65], it seems that the structure of α-Syn fibrils has been resolved. However, other reports have made this puzzle more complicated.

## 4 Evidences for α-Synuclein fibril polymorphism

Polymorphism describes the structural diversity present among amyloid fibrils generated from a specific polypeptide chain. This polymorphism within a sample can be detected whether the fibrils were formed *in vitro* or sourced from patients or diseased animals. Altering the conditions of fibril formation influences the range of morphologies that can be produced *in vitro*, and distinct fibril morphologies may contribute to the development of various amyloid disease variants *in vivo* [66].

Different polymorphs can be differentiated by various parameters such as diameter, twist, the number of protofilaments that constitute a fibril [67], their response to limited proteolysis, their diffraction patterns in fibril analysis [21], and their structural information obtained through NMR or cryo-EM techniques [[68], [69], [70]]. The polymorphs can be structurally different in the manner of protofilament assembly. The following discussion will delve into the results of research conducted on the polymorphic nature of α-Syn fibrils by cryo-EM.

**Fig. 2:**
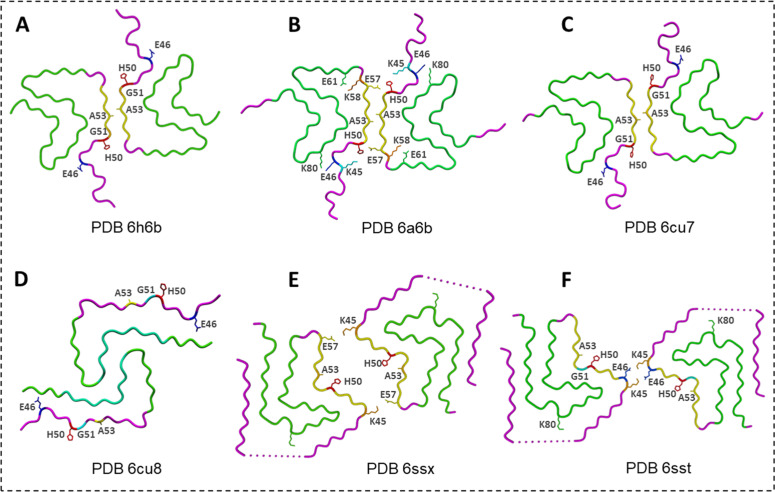
**α-Syn fibril polymorphs. (A)** C-terminal truncated α-Syn (residues 1–121) fibrils (α-Syn polymorph 1a) (PDB ID: 6h6b) [44]; **(B)** full-length α-Syn fibrillar structure (α-Syn polymorph 1a) (PDB ID: 6a6b) [65]; **(C)** α-Syn rod polymorph (straight, polymorph 1a) (PDB ID: 6cu7) [39]; **(D)** α-Syn twister polymorph (twisted and untwisted, polymorph 1b) (PDB ID: 6cu8) [39]; **(E)** α-Syn fibril polymorph 2a (PDB ID: 6ssx,6rt0) [42]; **(F)** α-Syn fibril polymorph 2b (PDB ID: 6sst, 6rtb) [42].

The first cryo-EM images with detailed structural analysis of C-terminal truncated α-Syn (residues 1–121) fibrils (α-Syn polymorph 1a) were obtained by Guerrero-Ferreira et al. [44] (Fig. 2A). This naturally occurring truncation enhances fibril formation, producing straight filaments typically 20–500 nm long. The ordered core consists of a compact β-sheet fold built from eight short, parallel β-strands, while both the N-terminal segment (residues 1–37) and the final ∼20 C-terminal residues remain flexible and disordered. A hydrophobic zone in the central region drives protofilament packing, forming a steric-zipper interface dominated by residues in the β_3_ region. This interaction yields a two-protofilament assembly whose interface is shaped largely by A53 and V55. These structural elements define the characteristic architecture of polymorph 1a and differentiate it from other α-Syn fibril forms.

Yaowang Li *et al*. [65] resolved the full-length α-Syn fibrillar structure (α-Syn polymorph 1a) (Fig. 2B) using cryo-EM, successfully constructing an atomic model for residues 37–99. Their findings revealed a left-handed helix with a helical pitch of 239 nm. Hydrophobic residues were folded inward, surrounded by hydrophilic residues on the surface. Residues 50–57 (^50^HGVATVAE^57^) from one protofilament created a dimer interface with opposing α-Syn subunits through a steric zipper, stabilized by hydrophobic interactions between A53 and V55. An electrostatic interaction was also observed between H50, K45, and E57 from the opposing subunit. Meanwhile, the two key electrostatic interactions between K58-E61 and E46-K80 were crucial for proper folding topology.

Binsen Li *et al*. [39] identified two additional dominant α-Syn polymorphs, referred to as twister (twisted, polymorph 1a) and rod (straight and untwisted, polymorph 1b) (Fig. 2**C and D**). Their different helical pitches (460 Å *vs.* 920 Å) reflect distinct core organizations. In the rod polymorph, only residues 38–97 were ordered and folded into a compact Greek-key-like motif, whereas the twister polymorph contained a shorter structured region (43–83) forming a bent β-arch enriched in branched hydrophobic residues. Despite their differences, both polymorphs share a set of conserved residues in the bends and turns and assemble symmetrically through hydrophobic steric-zipper interfaces located within the preNAC and NACore regions.

Conversely, Guerrero-Ferreira *et al*. [42] identified two additional dominant polymorphs of α-Syn fibrils, referred to as polymorphs 2a and 2b, which differ fundamentally from polymorph 1 structures (Fig. 2**E and F**). Their protofilaments contain two back-to-back β-arches and pair through salt-bridge interactions rather than steric zippers. In polymorph 2a, a K45–E57 salt bridge links the protofilaments, while in polymorph 2b the interface is stabilized by K45–E46 contacts. Hydrophobic interactions still dominate the packing, but polar contributions help shape protofilament geometry. As in earlier polymorphs, the N-terminal β-strand shows low density and remains disordered.

## 5 α-Synuclein fibril polymorphism in patient derived samples

Early reports by Spillantini *et al*. [51,71] first showed that α-Syn fibrils extracted from PD, DLB, and MSA brains adopt multiple polymorphs with distinct seeding capacities. Negative-stain EM revealed two main morphological classes: 10-nm twisted or straight filaments and a smaller population of 5-nm filaments. These features suggest that 5-nm protofilaments can pair to form wider twisted structures and that the molecules likely align parallel to the fibril axis.

**Fig. 3:**
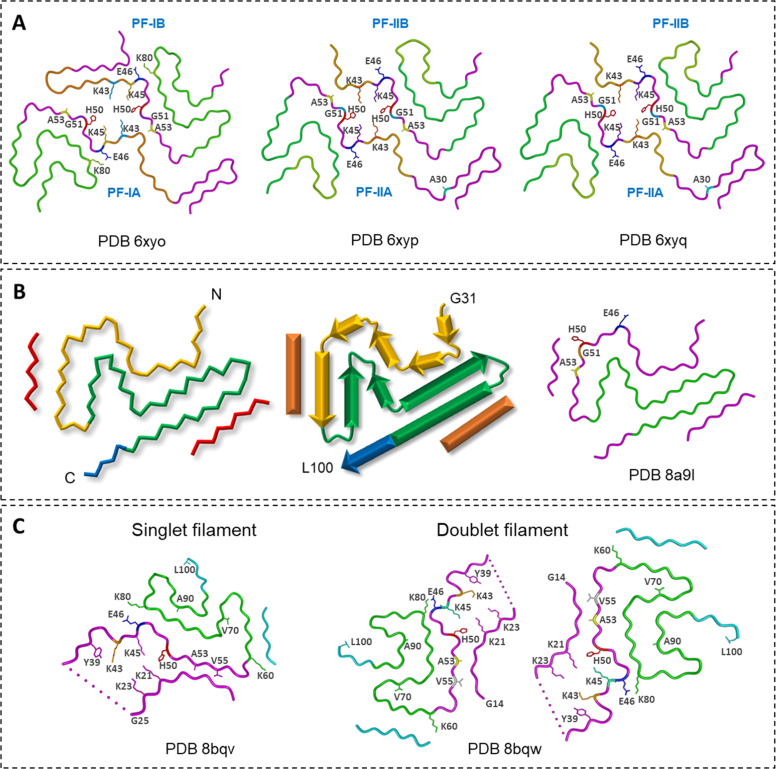
**α-Syn Lewy, MSA and JOS fold filaments. (A)** MSA folds; PDB ID: 6xyo for Type I filaments (MSA case 1), PDB IDs: 6xyp and 6xyq for Type II_1_ and Type II_2_ filaments (MSA case 2), respectively [72]. **(B)** Lewy fold (PDB ID: 8a9l) [73]. **(C)** Singlet and doublet filaments of JOS fold (PDB IDs: 8BQV and 8BQW).

More detailed structural insights were obtained by Schweighauser *et al*. [72], who used cryo-EM to identify two fibril types in MSA brain tissue (Types I and II) (Fig. 3A). Both shared an overall asymmetrical architecture built from two protofilaments with an extended N-terminal arm and a compact C-terminal body. The four protofilament variants (PF-IA, PF-IB, PF-IIA, PF-IIB) differed mainly in how their layered C-terminal motifs were arranged and in whether the E46–K80 salt bridge was tightly or loosely packed. These variations gave rise to distinct protofilament geometries while preserving a similar organizational theme.

A subsequent study from the same group resolved α-Syn filaments from PD, PDD, and DLB brains and identified a single dominant species termed the “Lewy fold” [73]. This right-handed assembly, built from residues 31–100 arranged into a three-layered β-sheet architecture, differed from the MSA folds [72] but shared a region of conformational similarity around residues 61–72 (Fig. 3B).

Structural analysis of brain material from a case of juvenile-onset synucleinopathy (JOS), caused by a duplication in SNCA, revealed yet another fibril architecture [74] (Fig. 3C). Despite the presence of a mutant 147-residue α-Syn, the fibrils adopted a compact left-handed fold composed largely of the wild-type core (residues 36–100). Most filaments (83 %) were single protofilaments, with a minority (17 %) forming C_2_-symmetric dimers. Their core conformation closely resembled features of MSA type I and II protofilaments, including a conserved E46–K80 salt bridge.

**Fig. 4:**
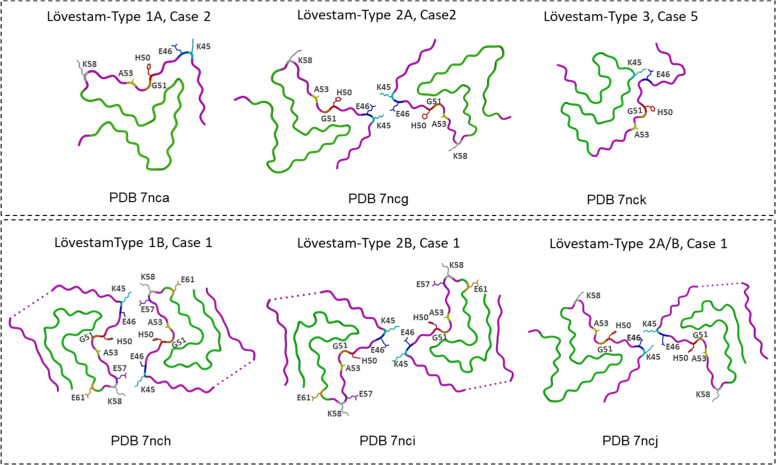
**α-Syn fibril polymorphs from *in vitro* seeded experiments with MSA cases-derived seeds.** Lövestam-type 1, 2, and 3 protofilaments along with folds A and B, were achieved through seeding activity using seeds from MSA cases 1, 2, and 5 [75].

In addition, Lövestam *et al*. [75] examined fibrils generated by seeding recombinant α-Syn with MSA-derived seeds. Remarkably, the resulting filaments did not replicate the exact seed structures; instead, they produced two main fibril types resembling the previously described 2a and 2b polymorphs. Differences stemmed mainly from their inter-protofilament salt-bridge patterns (E46–K58 in Lövestam-type 1; K45–E46 in Lövestam-type 2). Both exhibited two structural variants (folds A and B) with similar overall cores but slight local differences near the β-turn. A rarer “Lövestam-type 3″ filament, formed by a single protofilament and almost identical to MSA PF-IIB, appeared in one case and was thinner and more flexible than Lövestam-types 1 and 2 (Fig. 4) [42,75].

**Fig. 5:**
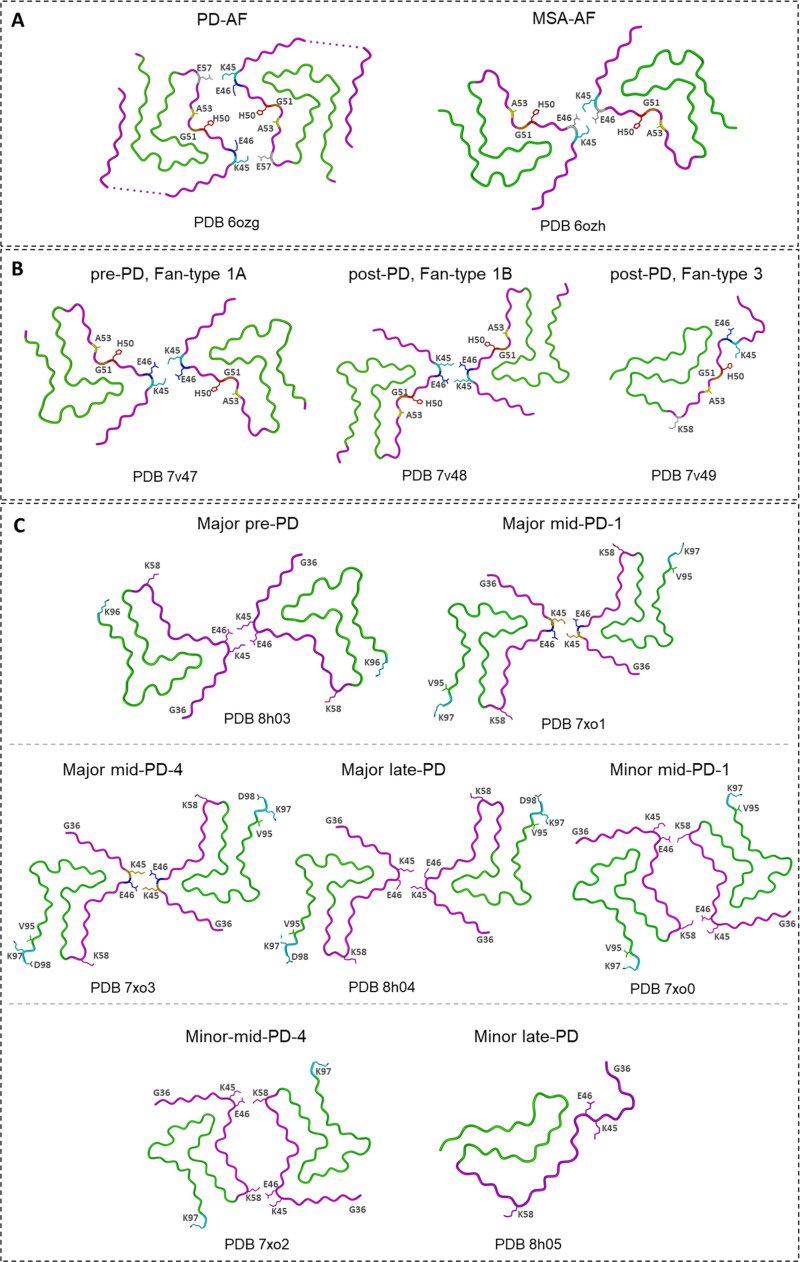
**Amplified α-Syn fibril polymorph with patient-derived seeds. (A)** Amplified α-Syn fibrils from PD and MSA brains [76]. **(B)** Amplified α-Syn aggregates from CSF of pre-PD and post-PD patients [78]. **(C)** Amplified α-Syn fibril polymorph from CSF of pre-PD, mid-PD, and late-PD patients [79].

Building on prior seeding investigations, Frieg *et al*. [76] analyzed the seeding activity of α-Syn fibrils extracted from PD and MSA brain tissue and found that, although both fibril types shared the same protofilament fold, their inter-protofilament interfaces and helical symmetries differed (Fig. 5**A**). PD-amplified fibrils (PD-AF) showed C_2_ symmetry, whereas MSA-amplified fibrils (MSA-AF) adopted 2₁ screw symmetry. These differences arose from distinct salt-bridge networks at the interface (K45–E57 in PD-AF; K45–E46/E57 in MSA-AF). MSA-AF also showed greater potency in seeding oligodendroglial α-Syn. Overall, both amplified fibrils closely resembled the previously described 2a and 2b polymorphs (Fig. 2**E** and **F**) [42,77], as well as *ex vivo* MSA structures.

Following this line of research, Yun Fan et al. [78], extended this analysis to α-Syn fibrils amplified from cerebrospinal fluid (CSF) of individuals at preclinical and postmortem PD stages (Fig. 5**B**). Pre-PD CSF was dominated by a Fan-Type 1A polymorph (85 %) structurally similar to polymorph 2b (Fig. 2**F**) [42], except that its N-terminal residues 14–25 were flexible and unresolved. Post-PD samples contained a related dimeric Fan-Type 1B polymorph (72 %) and a single-protofilament Fan-Type 3 polymorph (28 %), the latter displaying a fold reminiscent of polymorph 1a and sharing features with *ex vivo* MSA fibrils [72]. These findings suggest a structural shift in fibril architecture as PD progresses, with late-stage assemblies converging toward MSA-like folds.

The same group [79] later resolved α-Syn fibrils amplified from CSF at multiple PD stages—pre-PD, two mid-PD timepoints, and late-PD (Fig. 5**C**) [92]. All samples contained a dominant major polymorph, structurally conserved across disease stages, paired through K45–E46 salt bridges similar to polymorph 2b (Fig. 2**F**, PDB ID: 6sst). The minor polymorphs varied more substantially: mid-PD samples contained a polymorph resembling polymorph 2a (Fig. 2**E**, PDB ID: 6ssx), stabilized by E46–K58 salt bridges, while late-PD CSF yielded a distinct single-protofilament fold with similarities to polymorph 1a (Fig. 2**B**, PDB ID: 6a6b). These observations emphasize that while one structural motif persists throughout disease progression, secondary fibril populations diversify and may reflect stage-specific aggregation environments.

## 6 Effects of post-translational modifications on α-Synuclein fibril polymorphism

α-Syn undergoes various post-translational modifications (PTMs) that influence its aggregation and fibril formation. These modifications give rise to diverse fibril polymorphisms, each with distinct structural and functional properties.

### 6.1 Truncated α-Synuclein

**Fig. 6:**
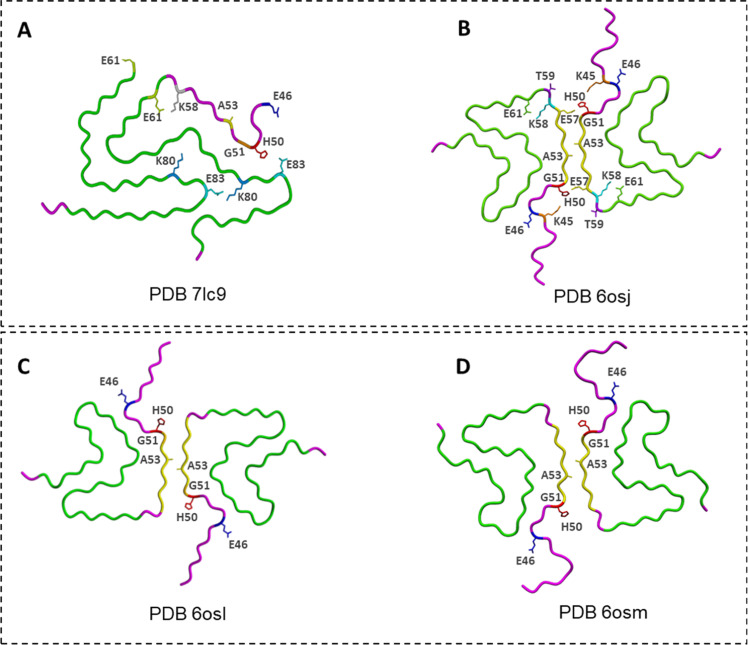
**Cryo-EM structures of α-Syn truncations. (A)** ΔN (41‒140) α-Syn N-terminal truncation [80]. **(B)** α-Syn full length (Ac1–140), **(C)** ΔC (Ac1–122), and **(D)** ΔC (Ac1–103) α-Syn C-terminal truncations [81].

McGlinchey *et al*. [80] showed that removal of the N-terminal region produces a highly homogeneous ΔN(41–140) fibril population with a compact, two-chain architecture (Fig. 6**A**). The truncated chains adopt different β-rich motifs that pair through a stabilizing intermolecular salt bridge, illustrating how N-terminal deletions reshape protofilament interfaces.

Complementary work by Ni *et al*. [81] resolved the fibril structures of three N-terminally acetylated (Ac) α-Syn constructs: full-length (Ac1–140) and two C-terminal truncations commonly observed in LBs (Ac1–122 and Ac1–103). They demonstrated that common C-terminal truncations (Ac1–122, Ac1–103) (Fig. 6**B, C** and **D**) progressively narrow fibril width but preserve the central steric-zipper motif formed by residues 50–57. Acetylation at the N-terminus promotes formation of an internal K58–E61 salt bridge that alters local β-arch geometry, leading to subtle but meaningful differences between full-length and truncated fibrils.

### 6.2 Phosphorylated and glycated α-Synuclein

One of the hallmark histological features of LBs and LNs is the accumulation of phosphorylated α-Syn fibrils. Elevated levels of α-Syn phosphorylation, including pY39, pS87, and pS129, are observed in the brains of PD and MSA patients. While pY39 and pS129 enhance α-Syn transmission and pathology, pS87 inhibits α-Syn aggregation and neurotoxicity [82,83].

**Fig. 7:**
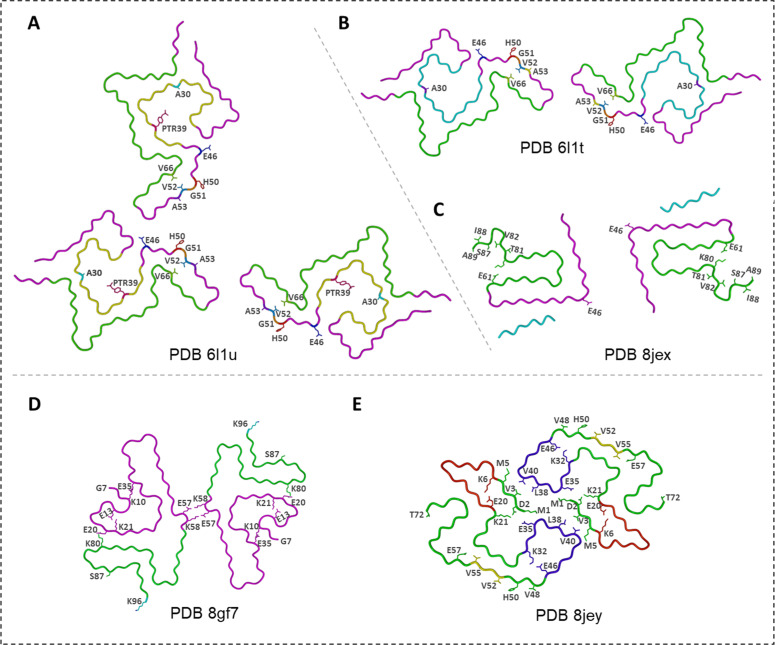
**Cryo-EM structures of phosphorylated and glycated α-Syn fibrils. (A)** and **(B)** The twist-trimer (PDB ID 6l1u) and twist-dimer (PDB ID 6l1t) polymorphs of α-Syn fibrils phosphorylated at Y39 (pY39), respectively [84]. **(C)** and **(D)** α-Syn fibrils polymorphs with O-GlcNAc modification at S87 (gS87) resolved by Hu (PDB ID 8jex) [83] and Balana (PDB ID 8gf7) [85], respectively. **(E)** α-Syn fibrils phosphorylated at S87 (pS87) (PDB ID 8jey) [83].

Phosphorylation at Y39 (pY39) generates one of the most structurally distinct α-Syn fibrils known. Zhao *et al*. [84] identified dimeric and trimeric twisted polymorphs with an unusually large fibril core extending from residues 1–100 (Fig. 7**A** and **B**). A network of electrostatic interactions around the phosphorylated tyrosine reorganizes the N-terminal region into a compact hook-like architecture and creates a unique hydrophilic channel at the fibril center. This structure cannot be replicated by wild-type α-Syn alone, underscoring the potent structural influence of Y39 phosphorylation [84]. Furthermore, cryo-EM analysis of cross-seeded fibrils formed by pY39 and WT α-Syn revealed the polymorph 1a structure (Fig. 4), demonstrating that WT α-Syn alone cannot readily adopt the pY39-like fibril structure.

Modifications at S87 yield a different outcome. α-Syn undergo O-GlcNAcylation, which can prevent amyloid aggregation. O-GlcNAcylation (gS87) stabilizes an “iron-like” fibril fold in which the sugar moiety forms integral interactions within the core, producing protofilaments that run side-by-side without tight interfacial packing (Fig. 7**C**) [83,85]. Phosphorylation at the same site (pS87) produces still another architecture: an extended arch-like fold in which the entire N-terminus becomes ordered, and protofilaments assemble through a combination of salt bridges and hydrophobic contacts not observed in WT fibrils. In both gS87 and pS87 fibrils (Fig. 7**D** and **E**), these rearrangements diminish toxicity and propagation capacity relative to unmodified fibrils, whereas pY39 has the opposite effect.

Together, the PTM-dependent fibril structures highlight how specific chemical modifications—whether removing termini, adding acetyl groups, or incorporating phosphate or GlcNAc groups—redirect α-Syn toward distinct folding pathways and biological behaviors [[83], [84], [85]].

## 7 Effects of mutation on α-Synuclein fibril polymorphism

Once the architecture of wild-type α-Syn fibrils was established, attention shifted to the hereditary mutations (E46K, A53T/E, G51D, and H50Q) that cluster at the protofilament interface [65,86]. Their shared location turns out to be crucial: each mutation subtly rewires the contacts that stabilize WT protofilaments and pushes the protein toward distinct assembly pathways.

**Fig. 8:**
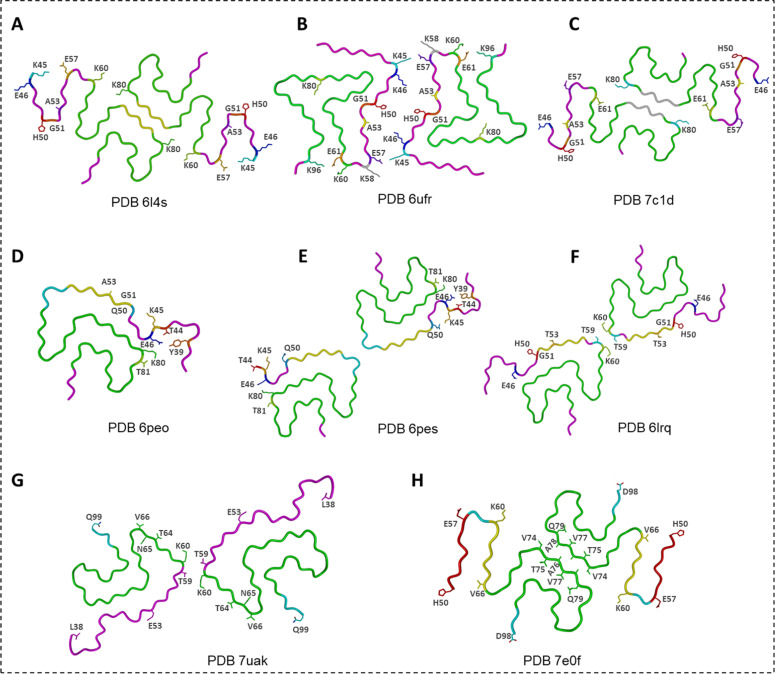
**Cryo-EM structures of mutated α-Syn fibrils. (A)** Ac-E46K α-Syn fibril [87]. **(B)** E46K α-Syn fibril [88]. **(C)** hE46K cross-seeded hWT α-Syn fibril [89]. **(D)** and **(E)** H50Q narrow and wide α-Syn fibrils, respectively [90]. **(F)** Ac- α-Syn A53T fibril [91]. **(G)** A53E α-Syn fibril [92]. **(H)** G51D α-Syn fibril [93].

The **E46K** mutation is a clear example (Fig. 8A) [87]. Wild-type fibrils rely on an E46–K80 salt bridge that helps anchor the N-terminal region into the fibril core. Substituting lysine at position 46 disrupts this anchor and forces a rearrangement of charges around residues 45–62. The resulting fibrils adopt serpentine folds in which the N-terminal segment becomes more flexible and the protofilament interface is rebuilt through alternative K45–E57 and K80–E61 interactions. A second structural study revealed [88] a different E46K polymorph with yet another packing scheme, tighter electrostatic “zippers,” and a distinct helical geometry, illustrating how a single mutation can open multiple folding routes. Both structures differ substantially from WT polymorphs, and both are consistent with the enhanced aggregation and pathogenicity observed experimentally (Fig. 8B). Cross-seeding experiments show that WT monomers can be templated into the E46K-like fold, confirming that this mutant strain is structurally transmissible (Fig. 8C) [89].

The **H50Q** mutation generates two unusual polymorphs—one narrow and one wide (Fig. 8**D** and **E**) [90]. The narrow fibril consists of a single protofilament; the wide fibril pairs two slightly different protofilaments. These structural variants share an extended β-arch but assemble in distinct ways, underscoring how even a conservative substitution at position 50 perturbs the interface and promotes architectural diversification. Table 1 shows the differences between protofilaments A and B.

**Table 1:** Differences between protofilaments A and B [90].

Protofilament A	Protofilament B
Includes residues G36–Q99	Includes residues T44–K97
Ordered β-arch structure via residues 36–46	Disordered region after T44
E46 participates in the Y39–T44–E46 hydrogen-bond network, and K80 forms a hydrogen bond with T81	E46 hydrogen bonds with K80
A hydrogen bond between Q50 and K45 supports the formation of an ordered β-arch	Absent in protofilament B

Mutations at position 53—**A53T** and **A53E**—were among the earliest linked to familial PD. Both lie at a critical segment of the protofilament interface [44,65,86]. A53T fibrils maintain the Greek-key-like fold found in WT but display a strikingly reduced protofilament interface, rendering the fibril less stable (Fig. 8F) [91]. This loosened packing is consistent with the high seeding efficiency of A53T fibrils. A53E produces an even more minimal interface and a more relaxed core geometry (Fig. 8G) [92]. Its fibrils incorporate additional peripheral densities (“islands”) that may interact with the β-arch and help compensate for the weakened core. These adjustments mark A53E as one of the most structurally distinct α-syn variants.

The **G51D** mutation produces fibrils associated with especially aggressive disease [94]. In the WT polymorph 1a, residues 50–57 form a key steric zipper. Replacing glycine 51 with aspartate disrupts this zipper and forces the region into a different β-hairpin-like arrangement (Fig. 8H) [95]. The fibril compensates by forming a new steric-zipper interface around residues 74–79 and by reorganizing the contacts between residues 45–57. The N-terminal portion (1–49) becomes disordered, and the resulting fold is looser and smaller than its WT counterpart. Even so, parts of the core resemble elements of WT polymorph 1a (PDB ID 6a6b) [44] and E46K fibrils (PDB ID 6l4s) [96], showing how mutation-induced detours can still converge on familiar structural motifs.

Altogether, the hereditary mutants reveal the sensitivity of α-Syn assembly to single-residue changes at the protofilament interface. Each mutation alters the balance of electrostatic contacts, steric-zipper interactions, and N-terminal packing, guiding monomers into strain-specific folds that parallel their divergent clinical behaviors.

## 8 Importance of α-Synuclein fibril polymorphisms

Various strain properties of seeds may explain the heterogeneity of synucleinopathies by differentially responding to cell populations and brain regions that are selected to be susceptible or resilient to the pathogenic properties of different seeds [97]. Evidence increasingly points to the possibility that prion strain-like phenomena may occur in neurodegenerative diseases like AD and PD. Structural variations at the molecular level in Aβ, tau, or α-Syn fibrils could be associated with, or even responsible for, differences in severity, symptomatology, progression, and neuropathology in these diseases. These effects could potentially arise from variations in the inherent cytotoxicity of different fibril polymorphs. This might be due to differences in the identities and conformations of surface-exposed amino acid sidechains, their capacity to bind metal ions that play roles in oxidative damage [98] or inflammation [99], their efficiency in self-propagation within tissues (perhaps due to varying susceptibility to fragmentation by biological processes), or other chemical, mechanical, or biological properties [100]. Therefore, it would be valuable to link structural models of α-Syn fibrils with their pathogenic counterparts, which may possibly imply difference in cellular effects, and bridge the gap between structures and neuronal toxicity.

Synucleinopathies exhibit significance clinical and pathological heterogeneity, characterized by disease-specific variations in clinical manifestations, progression rates, and the specific brain regions and cell types susceptible to α-synuclein accumulation and subsequent cellular degeneration [27,101]. Cerebral inclusions of α-Syn vary across synucleinopathies; PD and DLB are characterized by neuronal LBs and Lewy neurites (LNs), whereas MSA is marked by cytoplasmic α-Syn aggregates in oligodendrocytes [24]. Moreover, synucleinopathy samples demonstrate distinct seeding abilities in both *in vitro* and *in vivo* studies, with MSA-derived α-Syn aggregates showing greater potency in inducing α-Syn pathology compared to those from PD [26,34,102,103]. Distinct α-Syn pathologies can be observed following the injection of MSA brain extracts into different transgenic mouse lines [104].

Beyond the structural differences that result in unique fibril morphologies, research indicates that these polymorphs may display varying levels of toxicity in cellular studies [20,21]. Given the toxicity profiles observed in different amyloid polymorphs, an increasing array of studies is examining the similarities between amyloid polymorphism and the strain-like properties found in prions [20,21,25]. These polymorphs, much like prions, demonstrate nucleation (nucleated polymerization behavior), templating (seed aggregation processes), and intercellular transfer capabilities [[20], [21], [22],^25^,^105^,^106^]. Studies on transmission of MSA pathology in a transgenic mouse model with complete sets of clinical and biochemical markers provide compelling evidence for the prion-like behavior of α-Syn [[107], [108], [109]].

There are indications that different α-Syn strains give rise to the onset of distinct synucleinopathies. Peelaerts et al. [25] explored how different structural forms of α-Syn contribute to synucleinopathies such as PD and MSA. They identified α-Syn strains—fibrils and ribbons—that induce distinct pathological outcomes in mouse models. Fibrils were found to be the most toxic, causing significant neurodegeneration and motor impairments, while ribbons promoted the formation of LB-like deposits. α-Syn strains exhibited unique seeding capacities, resulting in strain-specific neurotoxic phenotypes. This research underscores the role of α-Syn strain conformation in disease progression and highlights the importance of considering these structural variations in understanding synucleinopathies.

Peng *et al*. [26] demonstrated that pathological α-Syn in glial cytoplasmic inclusions (GCIs) and LBs—referred to as GCI-α-Syn and LB-α-Syn, respectively—exhibit significant conformational and biological differences. GCI-α-Syn forms more compact structures and is roughly 1000 times more potent in seeding α-Syn aggregation compared to LB-α-Syn, reflecting the highly aggressive progression of MSA. Their findings also revealed that oligodendrocytes, unlike neurons, transform misfolded α-Syn into a GCI-like strain, underscoring that different α-Syn strains are shaped by unique intracellular environments. Furthermore, GCI-α-Syn retains its high seeding capacity when propagated in neurons, indicating that α-Syn strains are influenced by both the misfolded seeds and their intracellular contexts.

Structural differences in α-Syn fibrils could drive distinct pathological and progression pathways, influenced by specific post-translational modifications such as phosphorylation and ubiquitination [110]. For instance, C-terminal modifications are associated with different levels of proteasome inhibition [111]. Moreover, α-Syn expression varies among brain regions and cell types [112,113], with the unique cellular milieu of oligodendrocytes possibly playing a pivotal role in MSA. Furthermore, evidence suggests that α-Syn can form multiple structural polymorphs within a single disease [114].

α-Syn aggregate strains yield different clinical and pathological outcomes when introduced into transgenic mice [24]. In this study, each α-Syn strain showed a specific neurological symptom, timing of onset, and aggregate morphology—either globular or thread-like—that distinguish these diseases. Additionally, each strain showed selective affinity for certain brain cell populations, reflecting the patterns of cell-type susceptibility seen in human synucleinopathies. This suggests that recombinant or brain-derived α-Syn strains trigger distinct motor, biochemical, and pathological profiles in TgM83 mice, with each strain selectively impacting various brain regions and cell types [24].

De Giorgi *et al*. [115] observed that, in addition to traditional ThT-positive fibrils, α-Syn fibrillization in saline conditions also produces ThT-invisible polymorphs (τ^-^). The generation of τ^-^ fibril polymorphs is stochastic and can affect the apparent fibrillization kinetics detected by ThT. These polymorphs have been overlooked or mistaken for fibrillization inhibitions or failures. Structurally unique, τ^-^ polymorphs exhibit enhanced self-replication in cortical neurons. When injected into the substantia nigra pars compacta in mice, they induce synucleinopathy with extensive spread to regions such as the dorsal striatum, unlike ThT-positive fibrils, which have limited propagation and are cleared more rapidly by neurons. This suggests that fewer ThT-positive polymorph seeds would remain intracellularly to trigger secondary fibrillization [110]. hNH ssNMR analysis further confirmed that ThT-positive and τ^-^ polymorphs are distinct types of amyloid. ThT-positive polymorphs correspond to the “type 2″ fibril polymorph species [42,55]. However, the τ-polymorph did not match any of the previously characterized polymorph structure [37,39,42,[54], [55], [56]] and share “ThT invisibility” with ribbon-type amyloids assembled under low-salt conditions [21,54]. These findings suggest that α-Syn can spontaneously form various amyloids within the same environment, where ThT-negative (τ^-^) polymorphs may dominate and promote aggressive neuronal spread [115].

Distinct brain regions may preferentially accumulate specific dominant α-Syn polymorphs, each exhibiting unique biochemical properties, toxicity profiles, and cell-type specificities. It remains unclear at which stage in amyloid fibril maturation structural differentiation becomes possible. The morphology that ultimately predominates in a solution is influenced by environmental factors affecting both nucleus formation and propagation [116,117]. A single protein sequence can adopt multiple conformations, leading to fibrils with diverse morphologies. This capacity to assume various amyloid forms seems to be an inherent property of the polypeptide sequence, resembling the strain-like behavior observed in prions [118].

Furthermore, α-Syn fibrils with different molecular structures and surface morphologies may interact differently with monomers, resulting in distinct (cross-) seeding activities [119]. Misfolded α-Syn aggregates with unique molecular conformations (strains) have been shown to induce the formation of specific tau strains *in vivo* [22]. Research also indicates that diverse seeding species contribute to the development of polymorphisms [20,[120], [121], [122], [123], [124]].

## 9 Physical principles underlying the α-Synuclein fibril polymorphism

Fibril polymorphism is evident through variations in inter- or intra-residue interactions, differences in the number and arrangement of amino acid residues forming protofilaments, and distinct packing and orientation of these structures [42,125,126]. This polymorphism can arise from protein aggregation under varying conditions, generating misfolded monomers and oligomers with unique conformational characteristics [127]. Factors such as buffer composition, salt concentration, temperature, and other environmental conditions significantly impact the morphology and biological activity of α-Syn fibrils formed *in vitro* [21,23,25,57,115,128], indicating that solution modifications alter the molecular interactions among polypeptide chains, leading to different fibril structures. Furthermore, as α-Syn aggregation is inherently stochastic [129], multiple fibril types may form even under identical conditions, similar to what has been observed in prion proteins [130].

During *in vitro* aggregation it is known that various environmental factors can influence both the rate and mechanism of amyloid fibril formation, leading to the generation of different types of α-Syn fibrils. These factors include temperature [[131], [132], [133]], agitation [134], protein concentration [135], ionic strength [[136], [137], [138], [139]], denaturant concentration [140], pH [141], liquide-surface interfaces [142] and macromolecular crowding [143]. They affect primary nucleation, elongation, secondary processes (such as surface-mediated nucleation and fragmentation), fibril length and stability, as well as the structure of final aggregates [144]. Specifically, changes in solution pH or ionic strength have been shown to alter fibril secondary structure and stability [[145], [146], [147]].

The morphology and aggregation dynamics of α-Syn aggregate are greatly influenced by pH and ionic conditions. Under neutral pH and quiescent conditions, primary nucleation and secondary processes like fragmentation and surface-assisted nucleation are undetectable and α-Syn aggregation may favor non-fibrillar structures, which could have implications for its biological and pathological roles [141,148]. However, at lower pH levels (4.0 and 5.0) or in the presence of salts (such NaCl or MgCl_2_) at pH 7.0, secondary nucleation rates increase significantly, altering the balance between nucleation and growth, leading to the rapidly formation of large, amorphous aggregates with reduced fibril formation capacity [141,148].

Filamentous α-Syn aggregates also exhibit different molecular structures depending on experimental conditions such as pH and salt concentration. Under low salt conditions, α-Syn filaments have a ribbon-type morphology, while a twisted morphology is observed at high salt conditions [21]. These two distinct filaments have been shown to exhibit different disease phenotypes in mouse models [25].

*In vitro* studies have demonstrated that the primary nucleation of α-Syn amyloid fibrils can be initiated at hydrophobic/hydrophilic interfaces through heterogeneous nucleation, resulting in parallel β-sheet structures. However, such interfaces have not yet been identified *in vivo*. Research by Camino *et al*. [149] revealed that α-Syn can also undergo self-assembly into amyloid aggregates via homogeneous nucleation, favoring an antiparallel β-sheet structure. This configuration represents the most stable amyloid form under limited hydration, such as within α-Syn droplets formed through liquid-liquid phase separation (LLPS). These findings highlight the critical role of hydration in modulating the free energy landscape of nucleation, influencing not only the initiation of amyloid formation but also the type of polymorph produced. The results are particularly relevant for *in vivo* α-Syn aggregation, where varying hydration levels across cellular environments such as near lipid membranes or within membraneless compartments, may drive the formation of distinct amyloid polymorphs through either heterogeneous or homogeneous nucleation. This hydration-dependent process could link the variety of α-Syn polymorphs to the onset of distinct neurodegenerative diseases, with age-related cellular dehydration possibly increasing the risk of amyloid formation [149].

Various studies have shown that different α-Syn monomer species can inherit intrinsic properties from amyloid seeds, including specific conformations and pathogenicity [150]. This suggests that the attributes imparted to α-Syn monomers by seeds may play a crucial role in the formation of distinct α-Syn strains [97].

Aguirre et al. [151] demonstrated that the conformational state of monomeric α-Syn influences fibril polymorphism: compact monomers lead to rod-like fibrils, while extended monomers result in twisted fibrils. Through NMR analysis, they identified a polar interaction between the NAC region and the C-terminal domain that facilitates monomer compaction. This compaction is modulated by the chemical environment, such as NaCl, Ca^2+^, or cellular components, which can alter the NAC/C-terminal interaction. Their model provides mechanistic insights into how behavior of C-terminal domain drives fibril polymorphism during aggregation.

Sidhu *et al*. [107] suggested two mechanisms that determine α-Syn fibril morphology: competitive growth between various polymorphs during fibrillization, followed by a slow maturation or annealing phase where fibrils stabilize into distinct forms.

Recent studies have challenged the traditional view that the plateau phase in fibrillization indicates equilibrium, instead suggesting that this phase involves ongoing structural rearrangement within fibrils over time [64,144,[152], [153], [154]]. Morphological analyses using AFM and EM during the plateau phase reveal the presence of polymorphic fibrils [38,155]. Detailed NMR studies further indicate that variations in α-Syn polymorphs result from differences in backbone conformation, electrostatic interactions, and salt bridges [21,38,43]. This morphological polymorphism likely stems from the multiple conformations accessible to the soluble, unfolded α-Syn monomer [107].

### 9.1 Mechanism of polymorphism

According to the energy landscape theory of protein folding, multiple conformations may arise due to a rugged, funnel-shaped energy landscape that permits several low-energy conformations (local minima) during the early folding stages [[156], [157], [158]]. These local energy minima may act as kinetic traps, stabilizing partially folded or misfolded states [159,160]. The accessibility of these local minima, corresponding to various amyloid conformations, is thought to be heavily influenced by both aggregation conditions and the specific protein sequence [141,144,148,[161], [162], [163], [164]].

The findings of Reynolds *et al*. [165] suggest a need to reevaluate the protein folding and aggregation energy landscape, especially regarding the off-pathway region where amyloid fibrils were previously assumed to be the thermodynamically most stable structures. This revision implies a landscape with multiple minima, positioning amyloid crystals as the ground state. However, for these amyloid crystals to be validated as the minimum energy state, they must be experimentally accessible—a challenge particularly for longer proteins, as entropy constraints reduce their accessibility.

Zhao *et al*. [166] investigated the misfolding mechanisms of three α-Syn fibrils—isolated from a diseased human brain, generated *in vitro* with cofactor-tau, and generated *in vitro* without a cofactor—using molecular dynamics (MD) and Steered MD simulations (PDB ID: 6XYP [72], 7L7H [167], and 6SST [42], respectively). By analyzing boundary chain dissociation, they found distinct dissociation paths among the three fibril types. In the human brain fibril, monomer-template binding initiates at the C-terminal, with misfolding progressing toward the N-terminal. In the cofactor-tau system, binding begins at residues 58–66 (β_3_ region) and proceeds through the C-terminal coil (residues 67–79) and other segments, while in the cofactor-free system, two paths emerge: monomer binding from either the N- and C-terminal (β_1_/β_6_) or from C- to N-terminal, mimicking the human brain system sequence.

The structural polymorphism of amyloid fibrils primarily originates from the varying number of protofilaments involved in their formation. Additionally, differences in protofilament orientation or distinct conformations within the protofilaments can further enhance this polymorphism. Consequently, the "molecular polymorphism" of amyloid fibrils specifically refers to variations in protofilament conformations [168]. Polymorphic fibril structures may be transient, altering as time progresses [169].

**Fig. 9:**
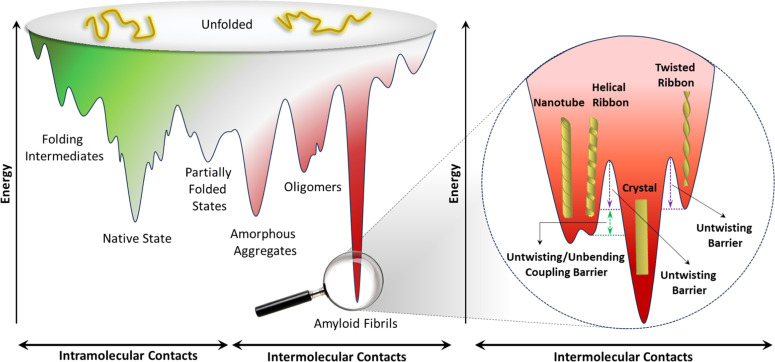
**The proposed energy landscape for the main amyloid polymorphs.** Amyloid crystals positioned at the ground state, while twisted ribbons, helical ribbons, and nanotubes are metastable states residing in relative energy minima. The figure is inspired by [170].

The twisted ribbon polymorph, commonly observed in amyloid fibrils, is considered a metastable state that can undergo two primary polymorphic transitions. In the first transition, the twisted ribbon transforms into a helical ribbon morphology as additional protofilaments progressively attach laterally. This structural change occurs when the width-to-thickness ratio reaches a critical threshold, a process explained through continuum mechanics frameworks. The helical ribbon may further stabilize by folding into nanotubes, thereby minimizing free energy through the release of line tension along the outer protofilament edges. In the second transition, the twisted ribbon undergoes thermal-induced untwisting, temporarily increasing its free energy before settling into a highly stable amyloid crystal form. This occurs through the lateral aggregation of untwisted protofilaments, which stack into large, translationally symmetric crystals with minimal surface area and interfacial energy, achieving a deep energy minimum in the folding landscape. Continuum mechanics, particularly the theory of chiral-filament elasticity, provides a solid model for understanding the observed transition from twisted ribbon to crystal, though the helical ribbon to crystal transition remains energetically unobserved due to inherent structural penalties (Fig. 9) [170].

Fibril polymorphism arises when protofilaments adopt distinct orientations, which in turn alter interaction surfaces, or when the polypeptide chains within protofilaments are organized differently. These distinct morphologies are believed to form due to the generation of structurally unique fibril nuclei, extending into diverse fibril forms [171]. This is supported by findings on the stochastic nature of fibril nucleation [172], as well as molecular dynamics simulations indicating that kinetic factors significantly influence the ensemble of fibril morphologies produced. Environmental conditions, such as temperature or solution composition, are critical in this process, as they modify the favorable interactions between polypeptide chains during fibril formation. However, understanding how specific solution conditions drive the formation of particular fibril structures remains limited. Within cellular contexts, factors like macromolecular crowding, molecular chaperones, and lipid bilayers can further modulate fibril morphology development, contributing to the complexity and variability observed in fibril structures [173,174].

The structure of amyloid fibrils is influenced by the molecular characteristics of their critical nuclei. This phenomenon, known as molecular-level polymorphism, suggests that for a polypeptide of a specific amino acid sequence, various nucleation events can lead to distinct critical nuclei and consequently different fibril morphologies [100]. Following nucleation, fibril growth can be modulated by conditions affecting processes like fragmentation. Fragmentation can be induced by mechanical shear forces (such as stirring or sonication) or biological tissue interactions, which increase the number of fibril ends and accelerate fibrillar mass accumulation. Notably, when fibril polymorphs coexist in early growth stages due to different nucleation events, the polymorph with the highest rate of fragmentation tends to dominate over time, as it produces more fibril ends, thereby increasing its growth rate and prevalence. This model illustrates how variations in nucleation and growth conditions lead to structural polymorphism in amyloid fibrils [100].

## 10 Concluding remarks

The clinical and pathological differences between synucleinopathies such as PD and MSA have been postulated to stem from unique strains of α-Syn aggregates, akin to what occurs in prion diseases. It has been realized over the last few years that polymorphism is present for many fibrillar proteins (*e.g.* in Aβ [40,[175], [176], [177]]) and also for α-Syn. The existence of polymorphs is an interesting phenomenon that has been linked to the phenomenon of strains in prions [178]. The fact that α-Syn spreads within an organism by cell-to-cell transmission, and the existence of different synucleinopathies [[179], [180], [181]] may possibly be linked to a prion-like behavior of the different polymorphs [[182], [183], [184]]. Polymorphism is manifested by differences in fibril morphology. Different polymorphs can have different biophysical properties and cellular responses and may be the structural bases of different strains in amyloids and prions [185]. Indeed, it has been observed over the last few years that sample preparation protocols, as well as the use of different NMR experiments and equipment yielding different signal-to-noise characteristics and different sensitivity to dynamic effects have led to a panoply of data that are disconcerting to compare [43]. α-Syn fibril polymorphism was first noted in patient samples but then supported by *in vitro* studies. After elucidation of the structural details from *in vitro* generated fibrils using cryo-EM, samples obtained from patients were also analyzed in more detail.

## CRediT authorship contribution statement

**Mitra Pirhaghi:** Writing – review & editing, Writing – original draft, Validation, Supervision, Project administration, Methodology, Investigation, Funding acquisition, Conceptualization. **Fatemeh Mamashli:** Writing – review & editing, Writing – original draft, Validation, Methodology, Investigation, Conceptualization. **Bagher Davaeil:** Methodology. **Mahya Mohammad-Zaheri:** Methodology. **Zahra Mousavi-Jarrahi:** Methodology. **Jörg Tatzelt:** Writing – review & editing. **Ali Akbar Saboury:** Writing – review & editing, Supervision.

## Declaration of competing interest

The authors declare that they have no known competing financial interests or personal relationships that could have appeared to influence the work reported in this paper.
